# Exploring perceptions of extreme environments and extremophiles in Chilean schoolchildren: an ethnographic study

**DOI:** 10.3389/fpubh.2024.1221731

**Published:** 2024-02-20

**Authors:** Mailing Rivera, Paola Fontana, Wilson Cortes, Cristian Merino, José Luis Vega

**Affiliations:** ^1^Facultad de Educación, Universidad de Antofagasta, Antofagasta, Chile; ^2^CIDSTEM, Instituto de Química, Pontificia Universidad Católica de Valparaíso, Valparaíso, Chile; ^3^Departamento Biomédico, Facultad de Ciencias de la Salud, Universidad de Antofagasta, Antofagasta, Chile; ^4^Departamento de Fisiologia, Facultad de Ciencias Biológicas, Universidad de Concepción, Concepción, Chile

**Keywords:** extreme environment, extremophiles, Atacama Desert, Patagonia, anthropological study

## Abstract

Chile is unique because of its diverse extreme environment, ranging from arid climates in the north to polar climates in Patagonia. Microorganisms that live in these environments are called extremophiles, and these habitats experience intense ecosystem changes owing to climate warming. Most studies of extremophiles have focused on their biotechnological potential; however, no study has examined how students describe extremophiles. Therefore, we were interested in answering the following question: How do schoolchildren living in extreme environments describe their environments and extremophiles? We performed an ethnographic study and analyzed the results of 347 representative drawings of participants aged 12–16 years from three schools located in the extreme environments of Chile San Pedro de Atacama (hyper-arid, 2,400 m), Lonquimay (forest, 925 m), and Punta Arenas (sub-Antarctic, 34 m). The social representation approach was used to collect data, and systemic networks were used to organize and systematize the drawings. The study found that, despite differences between extreme environments, certain natural elements, such as trees and the sun, are consistently represented by schoolchildren. The analysis revealed that the urban and rural categories were the two main categories identified. The main systemic networks were rural-sun (21,1%) for hyper-arid areas, urban-tree (14,1%) for forest areas, and urban-furniture (23,4%) for sub-Antarctic areas. When the results were analyzed by sex, we found a statistically significant difference for the rural category in the 7th grade, where girls mentioned being more rural than boys. Students living in hyper-arid areas represented higher extremophile drawings, with 57 extremophiles versus 20 and 39 for students living in sub-Antarctic and forest areas, respectively. Bacteria were extremophiles that were more represented. The results provide evidence that natural variables and semantic features that allow an environment to be categorized as extreme are not represented by children when they are focused on and inspired by the environment in which they live, suggesting that school literacy processes impact representations of their environment because they replicate school textbooks and not necessarily their environment.

## 1 Introduction

Chile is a unique country because of its diverse climates, ranging from arid or semi-arid climates in the Atacama Desert of northern Chile (22°–26°S) to sub-Antarctic or sub-polar climates in the Magellanic region of southern Chile (50–55°S) (1, 2). The Atacama Desert in northern Chile is the oldest and most arid nonpolar environment on Earth, exhibiting a unique combination of environmental extremes characterized by extreme dryness and high ultraviolet (UV) radiation levels (3). In a recent study, the highest levels of UV radiation on Earth’s surface were recorded at the Paranal Observatory and Chajnantor Plateau, both sites located in the Atacama Desert (4). Moreover, the regional climate in the Magellanic region is characterized by extreme events with high intensity and acute environmental effects (5). Organisms that live in these environments are called extremophiles and can live and grow under extreme conditions (6). Most studies on extremophiles have focused on their attractive biotechnological potential; their association with the processes of origin, evolution, and diversification of life on Earth; and their possible presence on other planets (6). However, no study has examined how students describe extremophiles. During the development of scientific knowledge, students must create new concepts and theories, mostly based on educational programs (7). However, children also build scientific knowledge through an intuitive understanding of the physical world based on their everyday experiences (8). This intuitive component could be predominant for students living in extreme environments. Here, we describe the perceptions of extreme environments and extremophiles among Chilean schoolchildren.

## 2 Materials and methods

### 2.1 Participants

A total of 347 participants aged 12–16 years participated in this study. The mean age was 12,13, 14- and 15-year-old for 7°, 8°, I°, and II grade, respectively. The three schools were state schools located in extreme environments of Chile: San Pedro de Atacama (hyper-arid, 22° 54’ S), Lonquimay (forest, 38° 27’ S), and Punta Arenas (sub-Antarctic, 53° 08’ S). Geographic sample sites were chosen because they were in three regions with different ecosystems and extreme climates in Chile. To do this, we resorted to the research network in extreme environments (NEXER) formed by the Universidad de Magallanes, Universidad de Antofagasta, and Universidad de la Frontera. The schools were state educational schools, with a vulnerability index of over 75%. The vulnerability index (IVE-SINAE) is an indicator risk of repetition and the of dropout rate and is defined by the Junta Nacional de Auxilio Escolar y Becas (JUNAEB), which provides nutrition to this population. The IVE-SINAE indicates the number of vulnerable students that the school has and from this data the vulnerability index of each commune is established. Moreover, Chile does not have private schools in all the communes as in the case of San Pedro de Atacama and Lonquimay since they are rural areas. The IVE-SINAE values described for the establishments in 2019 were Liceo Likan-Antai (San Pedro de Atacama) of 87.32%, Escuela Fronteriza (Lonquimay) of 93.06%, and Liceo Juan Bautista (Punta Arenas) of 75.53%. Written informed consent to participate in this study was provided by the participants’ legal guardian/next of kin. This study was approved by the Institutional Ethics Committee of the University of Antofagasta-Chile (N°050 /2017).

### 2.2 Procedure

The social representation approach was used to collect the data (9). This approach has enormous applicability in the understanding and interpretation of various social and human phenomena present in any cultural context (9). The design was developed using heuristic criteria that favor the description of the subjectivity of the participants conceived as producers of meaning (10). The students drew the environment they inhabited and, on the back, made a descriptive account of the drawing. The instructions were as follows: (1) On a horizontal sheet of paper, draw the environment in which you live. (2) Explain your drawings on the back of the sheet. (3) Use a new horizontal sheet of paper, fold the sheet into three columns or areas and place it on the drawing. (4) Each column is equivalent to a sector of the drawing. Imagine you have magic glasses to see the invisible or microscopic. (5) Place this second sheet on the drawing or in parallel. (6) Draw in the three areas of the drawing what you can see with your magic glasses. Drawing was used as a strategy to learn science and generate data (11) to increase citizens’ knowledge of extremophilic microorganisms. If science is imagined and drawn as a common human activity, it will be friendlier and more meaningful (12); for this reason, it is necessary to promote drawing as a school scientific activity (13). At this point, we remember Ramón y Cajal (1852–1934), who illustrated knowledge about the brain with drawings (14). Therefore, it is necessary and opportune to learn science by drawing to overcome, to a certain degree, the tendency to focus teaching on the descriptive accumulation of a particular phenomenon (15) and move towards the construction of a cognitive and discursive science (16), which recognizes production processes (17). These contributions are a call to think about strategies that compromise students’ cognitive activities, such as the use of drawings in science classes. Each activity, lasting 45–60 min, was performed individually and digitized. To do this, different drawings and descriptions were analyzed from the perspective of their contents to make the analytical components visible and emerge. The dimensions obtained were (1) information about the extreme environment and extremophiles, (2) the field of representation of the extreme environment and extremophiles, and (3) attitudes towards the extreme environment and extremophiles. Validation was sought from the categories of emerging analysis, recognition, and explanation provided by the students about their activities and interactions. Unlike quantitative methods, ethnographic research does not impose pre-defined categories for analysis. Instead, as we delved into the collected data from schools, distinct groups of perceptions about the environment and extremophiles naturally emerged. These groups were characterized by recurring themes, which we labeled as “emerging analysis categories. Congruence was sought, first, with the participation of schoolchildren in the categorical construction, second, by encouraging them to investigate their own actions, and finally, to develop interactive descriptions. Within the learning environment, students were actively engaged in a process of self-inquiry, investigating their own perceptions and representations of the environment and extremophiles. This involved activities such as observation, drawing, and verbal explanation, fostering critical thinking and analysis skills applicable both within the classroom and in their personal experiences. Observation and communication skills are part of the science curriculum. We postulate that these skills should be emphasized and enhanced in the classroom from an early age and throughout training, to complement functional and scientific literacy.

### 2.3 Data analysis

**Figure 1:**
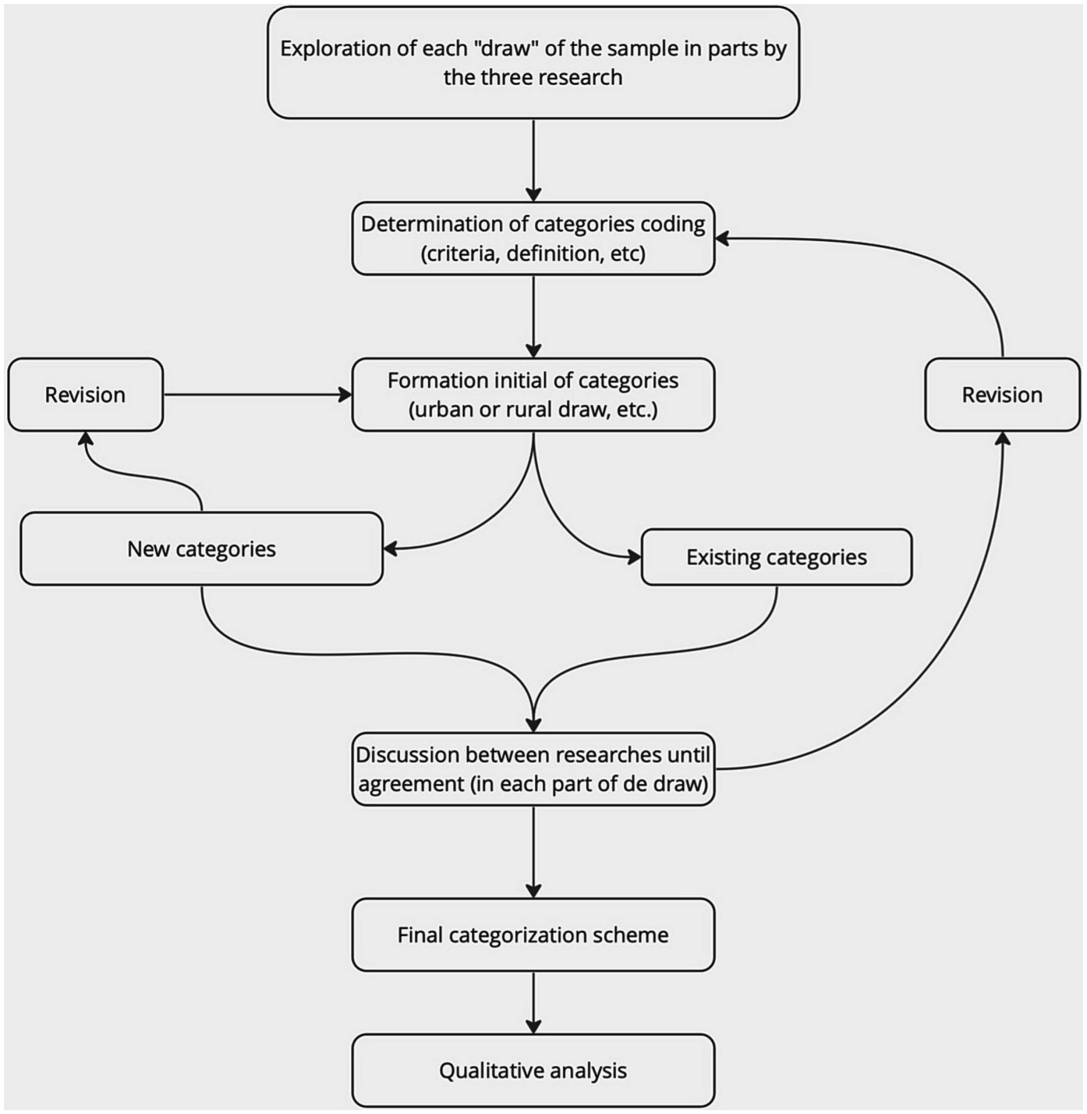
A schematic presentation of the procedure of analysis. A schematic presentation of systemic networks was adapted by Papageorgiou et al. (20).

Systemic networks have been used to organize and systematize drawings interested in the description and representation of the meaning of the semiotic resources of language (18). The analysis of the representations was developed in the following stages: (1) search for regularities and their representation through the conformation of systemic networks (18); (2) statistical description of the data, according to the higher frequencies of appearance of the representations; and (3) triangulation of the data obtained through drawings, speeches, and answers to brief interviews to answer the research question. It is important to mention that, when selecting drawings, the interpretation of the data is based on our objectives. The drawings were filtered based on researchers’ interests, preconceptions, and perceptions. No system of analysis can be conducted without the fact that the relationship between the drawings’ perceptions and their analysis is problematic. However, this type of analysis allows intuitive relationships to become explicit and therefore more accessible to discussion (13). The systematization of data from systemic networks allows us to visualize trends in drawings made by students (19). A schematic representation of the analytical procedure is presented in Figure 1. To validate the systemic networks, different experts (biologists, anthropologists, and professors) used the kappa index (concordance index). There are several proposed concordance indices, where the most reliable is the proportion of agreements observed, that is (a + b)/N, which is a very intuitive and easily interpretable index because it takes values between zero (total disagreement) and one (maximum agreement). However, as a reproducibility indicator, it has the drawback that even if the two observers are classified using independent criteria, a certain degree of agreement could eventually be produced by chance. It is desirable that a concordance index takes this fact into account and, in some way, indicates the degree of agreement that exists above that expected by chance. In this sense, the most used index is the one proposed by Cohen (equation 1), called the *kappa* index (k), which is defined as

$$k=\frac{P_{0}-P_{c}}{\;1-P_{c}}$$

P_0_ is the proportion of observed agreements, and P_c_ is the proportion of agreements expected in the hypothesis of independence between observers, that is, agreement by chance (21). In our case, the degree of agreement in the classification of the drawings in the systemic network was 0.8011, whereas according to the K value, in the case of the classification of the drawings, there would be a maximum agreement of 80.0 and 20.0% of that expected by chance between the pairs of experts, and 20% associated with chance. According to Cerda and Villarroel del (21), for values obtained in the range [0.61–0.80], the degree of agreement is considered substantial.

### 2.4 Statistical analysis

The results are expressed as the mean ± S.E.M., and statistical significance was set at *p* < 0.05. Because results do not fulfil the assumptions of parametric tests, we used a Wilcoxon rank-sum non-parametric tests to compare two conditions. Prism software (9.0 version, GraphPad Inc., California, United States) was used for the graphs and statistical analyses.

## 3 Results

### 3.1 Extreme environment and extremophiles representations

**Figure 2:**
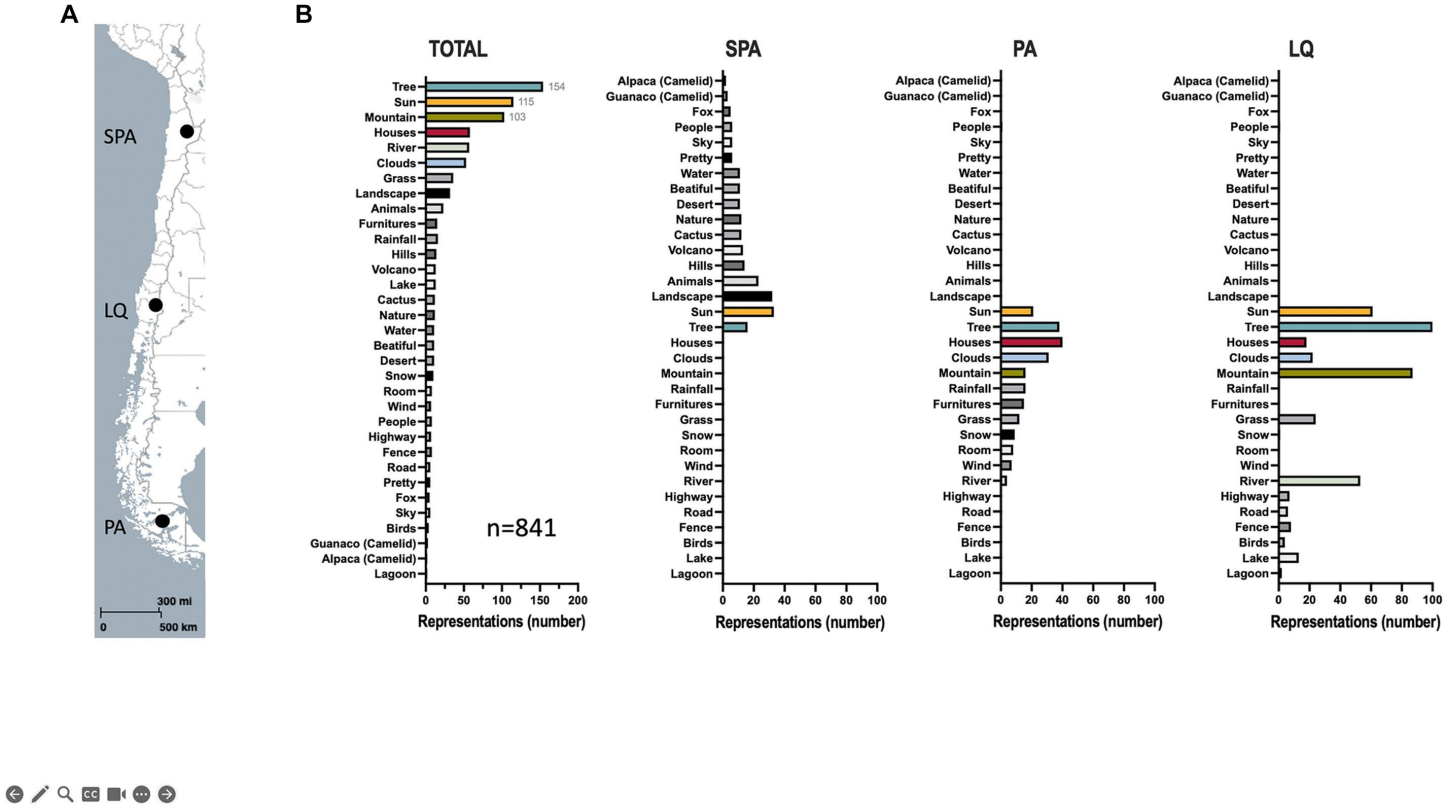
Representation of an extreme environment. **(A)** Locations of the selected Chilean schools that participated in the study are indicated on the map. **(B)** The most frequent representations are drawn for all (total) and different areas. SPA, San Pedro de Atacama; LQ, Lonquimay; PA, Punta Arenas. A map was created using MapCreator (MapCreator, United States).

**Figure 3:**
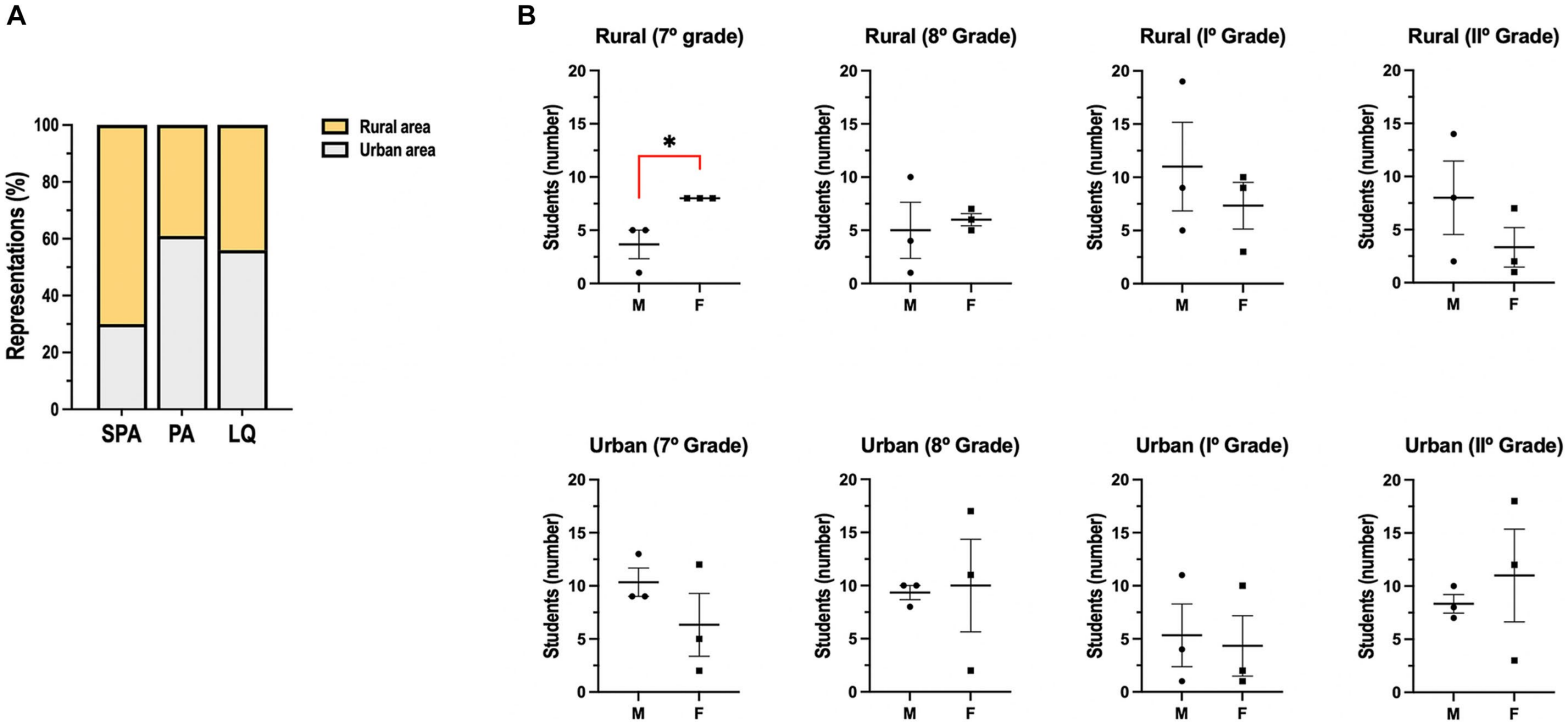
Distribution of rural and urban categories. **(A)** Percentage of rural (yellow) and urban (gray) categories from the three areas included in the study. **(B)** Urban and rural categories were separated by sex for each grade. SPA: San Pedro de Atacama, PA: Punta Arenas. ^*^p < 0.05 between male and females by Student’s *t*-test.

**Figure 4:**
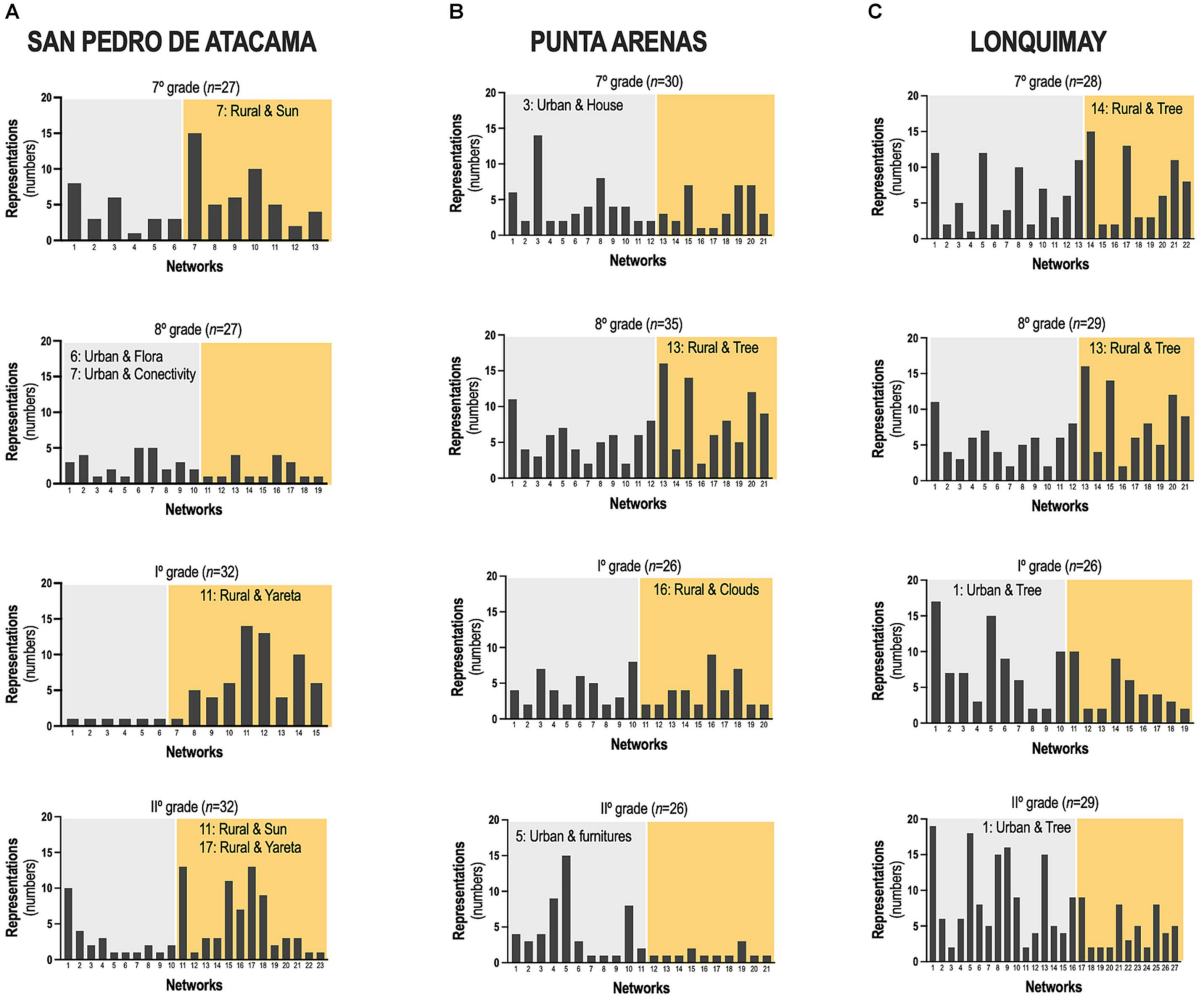
Identification of systemic networks. A total of 347 participants drew their environments from **(A)** San Pedro de Atacama, **(B)** Punta Arenas, and **(C)** Lonquimay. The most represented systematic network in each grade and the urban (gray) and rural (yellow) categories are indicated.

**Figure 5:**
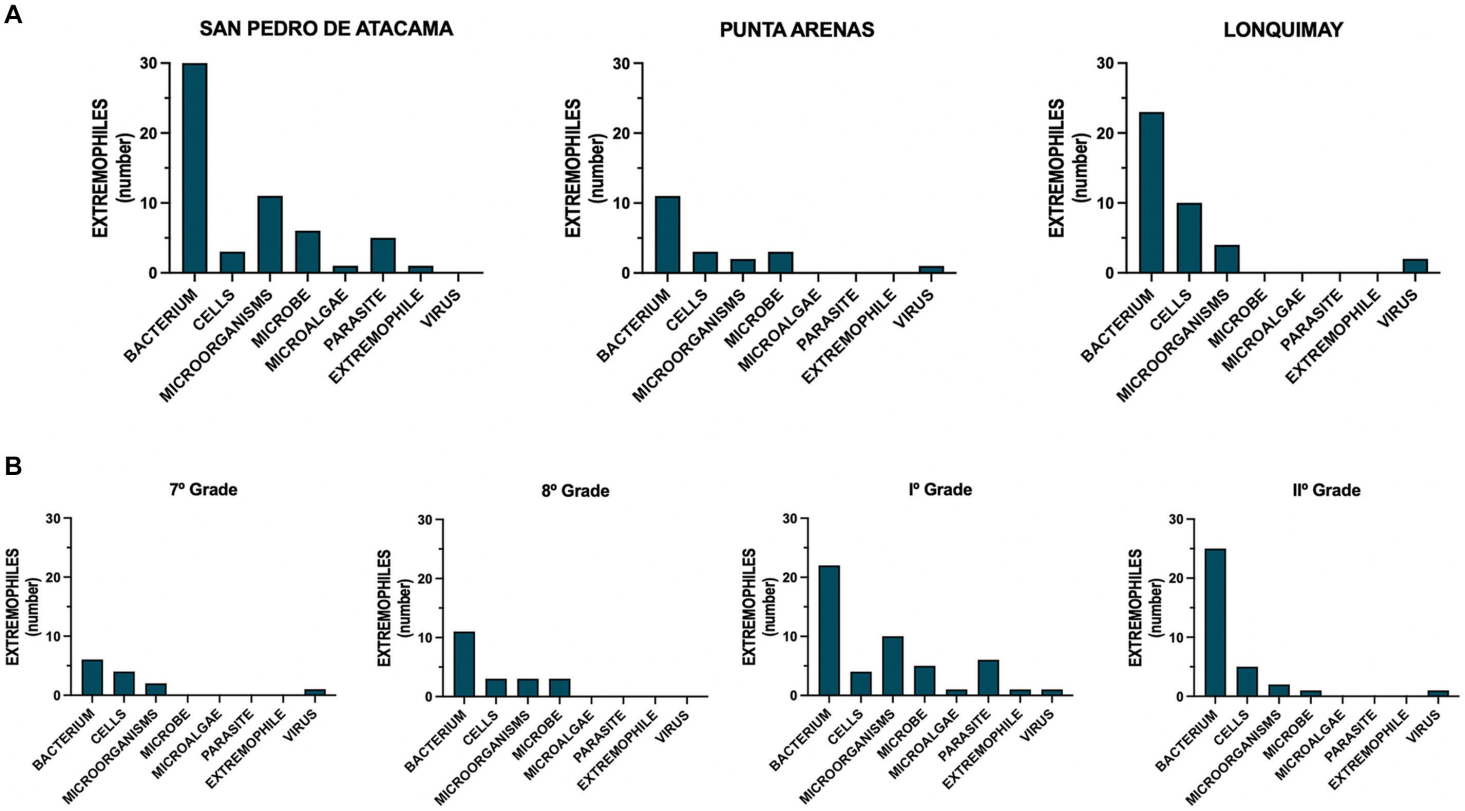
Identification of extremophiles. Distribution of the extremophiles drawn by the participants according to **(A)** area or **(B)** grade.

The three schools are geographically distinct. However, the results showed that children drew on similar elements (Figure 2). Trees, suns, and mountains were the most frequent, accounting for 18.3, 13.7, and 12.2%, respectively (Figure 2B, Total). The sun, landscape, and animals were the most frequent representations of San Pedro de Atacama (Figure 2B, SPA). Trees, houses, and clouds were the most frequent representations of the Punta Arenas (Figure 2B, PA). Trees, mountains, and the Sun were the most frequent representations of Lonquimay (Figure 2B, LQ). The analysis of systemic networks revealed that the urban and rural categories were the two main categories. San Pedro de Atacama’s presented a higher percentage of rurality with 70.0% (Figure 3A). When the results were analyzed by sex, we found a statistically significant difference for rural students in 7th grade (Figure 3B). Girls mentioned being more rural than boys did (Figure 3B). The specific analysis for grade in each school showed that for San Pedro de Atacama (*n* = 118), the most frequent network for seven grades was network #7 rural-sun (21.1%), for eight grades was network #6 urban-flora and network #7 urban urban-connectivity (11.1%), for grade I was network #11 rural-yareta (20.3%), and for grade II were network #11 rural-sun and network #17 rural-yareta (13.4%) (Figure 4A). For Punta Arenas (*n* = 117), the most frequent network for seven grades was network #3 urban-house (16.5%), for eight grades was network #13 rural-tree (11,4%), for grade I was network #16 rural-clouds (11,1%), and for grade II was network #5 urban-furnitures (23.4%) (Figure 4B). For Lonquimay (*n* = 112), the most frequent network for seven grades was network #14 rural-tree (10.6%), for eight grades was network #13 rural-tree (11.4%), and network #1 urban tree for grades I and grades II (14.1% and 9.8%, respectively) (Figure 4C). The data showed that students from San Pedro de Atacama represented most drawings of extremophilic microorganisms, with 57 microorganisms versus 20 for Punta Arenas and 39 for Lonquimay (Figure 5A). Details by area revealed that bacteria were the most abundant extremophiles (Figure 5A). Analysis by sex did not reveal differences in the description of extremophiles (data not shown). Finally, the I° grade contained the most extremophilic microorganisms (Figure 5B).

## 4 Discussion

The number of terms used to describe nature or the environment in which one lives varies significantly; however, despite these differences, certain objects prevail that have been attributed to perceptual salience (22). This study provides evidence that natural variables that allow an environment to be categorized as extreme are not represented by children when drawing the environment in which they live. In the three places studied, from the desert in San Pedro de Atacama to Patagonia in the south of the world, students from the 7th to 2nd grades represented trees, the sun, and mountains as the main elements when drawing their environment. Through the drawings, the students provided their positions regarding situations, events, objects, and people that surround them without establishing causal relationships. The results showed that schoolchildren from these communities did not show any differences in the places they inhabited. Other studies have addressed the question: Why do schoolchildren represent knowledge from science texts and not necessarily what they directly observe and experience? (8). One possible answer is that their social representations need a fundamental conceptual reorganization that originates from the suspension or revision of some presuppositions belonging to naive scientific theory (9). In this study, we identified the anthropological thresholds of Chilean schoolchildren regarding extreme environments and the microorganisms that inhabit these environments. This anthropological threshold represents one’s knowledge, experiences, beliefs, and opinions about extreme environments (7, 23, 24). For this reason, we postulate the need to recognize them in the processes of functional and scientific literacy; not doing this implies excluding cultural, social, and anthropological memory elements in the classroom (25). Regarding extremophiles, the data showed a scalar view that included one group of insects and worms; another group that included cells, bacteria, and viruses; and another group that included atoms. Bacteria are the most commonly mentioned extremophiles. From this perspective, students build explanations based on causal relationships between facts. However, they did not necessarily relate them to their primary characteristics, suggesting that the students perfectly adapted to their environments. Finally, this study highlights the importance of environmental representations as anthropological thresholds to understand extreme environments and extremophilic microorganisms. This emphasizes the need for greater awareness and appreciation of the physiological conditions of these organisms and their habitats to promote environmental preservation. We consider that didactic resources are required to highlight the physiological conditions of microorganism extremophiles that allow their survival in these habitats and to promote critical learning and attitudes toward preserving these environments.

## Data availability statement

The raw data supporting the conclusions of this article will be made available by the authors, without undue reservation.

## Ethics statement

The studies involving humans were approved by the Institutional Ethics Committee of the University of Antofagasta-Chile (N°050/2017). The studies were conducted in accordance with the local legislation and institutional requirements. Written informed consent for participation in this study was provided by the participants’ legal guardians/next of kin.

## Author contributions

MR, WC, CM, and JV drafted the manuscript. MR, PF, JV, and CM conducted the study in classrooms. All authors contributed to the article and approved the submitted version.
